# Massive Necrotizing Pancreatitis in an Immunosuppressed Renal Transplant Recipient (Successful Therapy)

**DOI:** 10.1155/1993/38478

**Published:** 1993

**Authors:** Joel W. Slaton, John M. Howard, Steven H. Selman

**Affiliations:** 1 Department of Urology and Department of Surgery Medical College of Ohio and The Toledo Hospital Toledo Ohio USA; 2 Harris McIntosh Tower 2121 Hughes Drive Suite 940 Toledo Ohio 43606 USA

## Abstract

Severe pancreatitis may be associated with massive necrosis of the pancreas and/or retroperitoneal adipose tissue. Toxicity results from the dead tissue and secondary infection. A 45 year old patient, while fully immunosuppressed, developed this complication following cadaveric renal transplantation. He survived continued immunosuppression, 16 operative debridements of the retroperitoneum, and maintained a functioning renal transplant.

In view of the previously reported high mortality rates from mild pancreatitis after transplantation, the current experience warrants further evaluation of the open method of treatment.


HPB Surgery, 1993, Vol. 7, pp. 157-164
Reprints available directly from the publisher
Photocopying permitted by license only

© 1993 Harwood Academic Publishers GmbH
Printed in the United States of America

### CASE REPORT

### MASSIVE NECROTIZING PANCREATITIS IN AN IMMUNOSUPPRESSED RENAL TRANSPLANT RECIPIENT (SUCCESSFUL THERAPY)

JOEL W. SLATON, JOHN M. HOWARD and STEVEN H. SELMAN
*Department of Urology and Department of Surgery, Medical College of Ohio and The Toledo Hospital, Toledo, Ohio, USA*

*(Received 31 August 1992)*

Severe pancreatitis may be associated with massive necrosis of the pancreas and/or retroperitoneal adipose tissue. Toxicity results from the dead tissue and secondary infection. A 45 year old patient, while fully immunosuppressed, developed this complication following cadaveric renal transplantation. He survived continued immunosuppression, 16 operative debridements of the retroperitoneum, and maintained a functioning renal transplant.

In view of the previously reported high mortality rates from mild pancreatitis after transplantation, the current experience warrants further evaluation of the open method of treatment.

KEY WORDS: Necrotizing pancreatitis, renal transplant, immunosuppression

### INTRODUCTION

Within the past decade, mortality rates from massive pancreatic and peripancreatic necrosis have been reported to be quite high — as high as 50--100% in earlier series. The mortality rate today is falling, although many patients develop a complicated and near fatal course. Most of the necrotic retroperitoneal tissue is now recognized as being necrotic adipose tissue, although portions of the pancreas may sometimes undergo necrosis[1,2]. The role of infection has been central to most discussions of management.

Acute pancreatitis is a well known complication of renal transplantation. The resultant mortality rates have been extremely high, even with pancreatitis of a mild or moderate degree of severity[3-5].

As an indication of progress in the management of such patients, the following report reflects the advances currently underway. The patient developed acute pancreatitis with massive retroperitoneal necrosis while immunosuppressed after renal transplantation, remaining immunosuppressed, and was discharged from the hospital with his transplant kidney still functioning.

Address correspondence to: John M. Howard, M.D., Harris McIntosh Tower, 2121 Hughes Drive, Suite 940, Toledo, Ohio 43606, USA. Telephone: (419) 479-2626 Fax No: (419) 479-6962

### CASE REPORT

A 45-year-old white male with end-stage glomerulonephritis underwent a cadaveric renal transplantation on January 15, 1989. Immunosuppression in the immediate postoperative period included prednisone 200 mg daily, subsequently tapered to 30 mg per day on postoperative day 19. Cyclosporine A, 14 mg/kg, was also administered. On the second postoperative day, the patient unexpectedly became oliguric. A renal ultrasound showed no evidence of obstruction, and a renal scan revealed unobstructed blood flow to the transplanted kidney. The overall picture was consistent with acute tubular necrosis. Consequently, the daily cyclosporine dosage was reduced to 5 mg/kg and azathioprine, 1.5 mg/kg daily, was added. Hemodialysis was begun the second postoperative day; the patient's serum calcium had declined to 5.8 mg/dl. Hypocalcemia persisted, falling to 4.3 mg/dl before being reversed by repeated intravenous infusions of calcium gluconate. The patient's serum calcium level stabilized by the seventh postoperative day at 7.2 mg/dl.

Gross hematuria developed on the seventh postoperative day. Significant laboratory findings included a prothrombin time of 28.3 seconds, a partial thromboplastin time of 41.9 seconds, a platelet count of 179,000, and a normal fibrinogen level. Azathioprine was discontinued and the patient was also treated with a transfusion of fresh frozen plasma. Oliguria had resolved by the eighth postoperative day.

On the 11th postoperative day, the patient complained of nausea and abdominal pain. At the same time, his white blood cell count increased to $20 \times 10^3$/mm. Serum amylase and lipase levels, measured for the first time, were normal. A voiding cystogram showed no evidence of urinary extravasation. An abdominal CT scan revealed a markedly enlarged pancreas with an extensive peripancreatic fluid collection. The patient was diagnosed as having acute pancreatitis. Initial treatment consisted of nasogastric suction and total parenteral nutrition. On the 17th postoperative day, the patient developed spiking temperatures to 39.2 °C. Blood cultures grew pseudomonas aeruginosa, for which the patient was started on Tobramycin and Ceftazidime.

On the 17th postoperative day, a repeat CT revealed progression of the peripancreatic necrosis with extension into the fat planes and soft tissues of the retroperitoneum (Figures 1a and 1b). The renal function remained stable (creatinine 1.7 mg/dl) on an immunosuppressive regimen of intravenous cyclosporine (5.5 mg/kg) and methylprednisolone (35 mg/day). A Hickman central venous catheter was inserted on the 24th postoperative day to permit prolonged parenteral nutrition. The transplant incision continued to drain purulent fluid. On postoperative day 35, severe diarrhea developed secondary to Cl. difficile. Tobramycin and Ceftazidime were discontinued, and metronidazole was started.

On the 38th postoperative day, spiking fevers recurred. Blood cultures demonstrated Pseudomonas. A repeat CT scan showed further extension of the inflammatory process into the retroperitoneum with the loss of the normal anatomical markings of the pancreas. Two fluid collections were identified, one 6.5 cm diameter and another 7.5 cm diameter were in or adjacent to the head and body, respectively. A CT-directed aspiration of peripancreatic fluid was performed. Twenty millimeters of thick, chocolate-colored fluid was obtained. Cultures of this material were positive for Pseudomonas.

The patient continued to manifest fever and leukocytosis. Exploration via a bilateral subcostal incision was performed on the 39th post-transplant day and

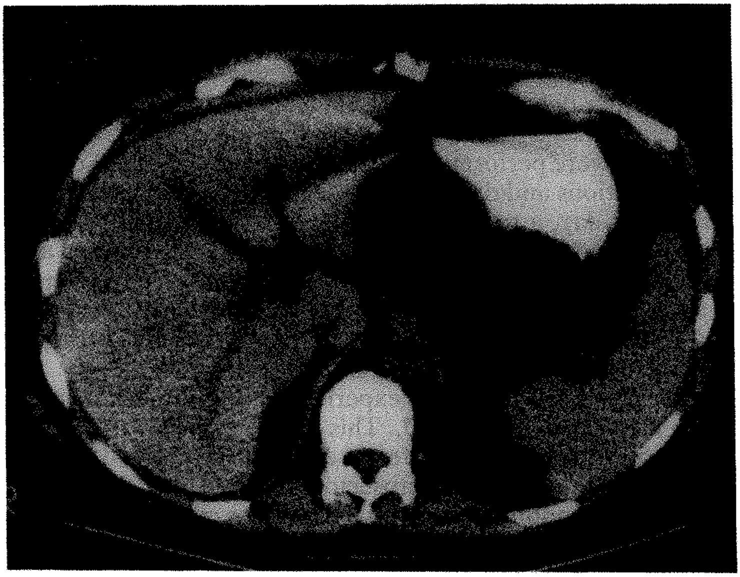

**Figure 1a** CT on 17th post-transplant day showing large retrogastric area of peripancreatic necrosis. Note gas bubbles in the necrotic debris.

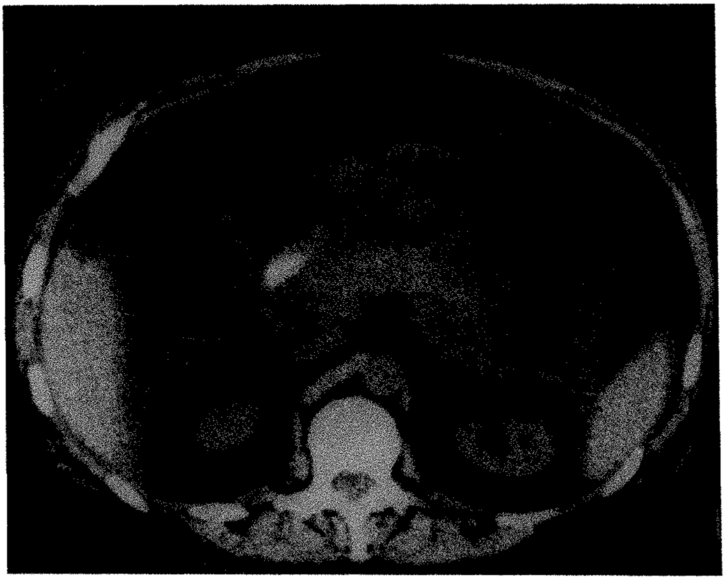

**Figure 1b** CT of the same date revealing extension of the necrotic mass into the mesentery of the intestine.

consisted of extensive peripancreatic necrosectomy (Figure 2) and open drainage of the peripancreatic abscess (Figure 3). Through a separate incision, a feeding jejunostomy tube was inserted. The necrosectomy wound was packed open in anticipation of further debridement. Tobramycin and Ceftazadime were restarted. In the subsequent 51 days, a total of 15 additional intraoperative dressing changes and necrosectomies were performed under general anesthesia. At this point, twice-a-day bedside irrigations and dressing changes were begun. On April 21, 1989, the 95th post-transplant day, a repeat CT scan revealed no further fluid collections.

Tobramycin and Ceftazidime had been discontinued two weeks after the initial drainage procedure. During the six weeks of intraoperative dressing changes, the patient was treated twice for staphylococcus epidermidis bacteremia with seven day courses of Vancomycin. Nutrition was maintained primarily through the jejunostomy, supplemented as necessary by parenteral nutrition.

Therefore, the patient continued to experience low grade fevers and intermittent drainage from the transplant wound. This was believed to represent egress of residual retroperitoneal tissue. His fever eventually resolved and by the 130th postoperative day, the pancreatic necrosectomy wound was clean and granulating. The transplant wound continued to drain a moderate amount of purulent material. The renal allograft continued to function well maintaining a serum creatinine of 1.1 mg/dl. Twelve months after transplantation, the kidney failed. Biopsy revealed advanced glomerulonephritis in the transplanted kidney. Chronic hemodialysis was

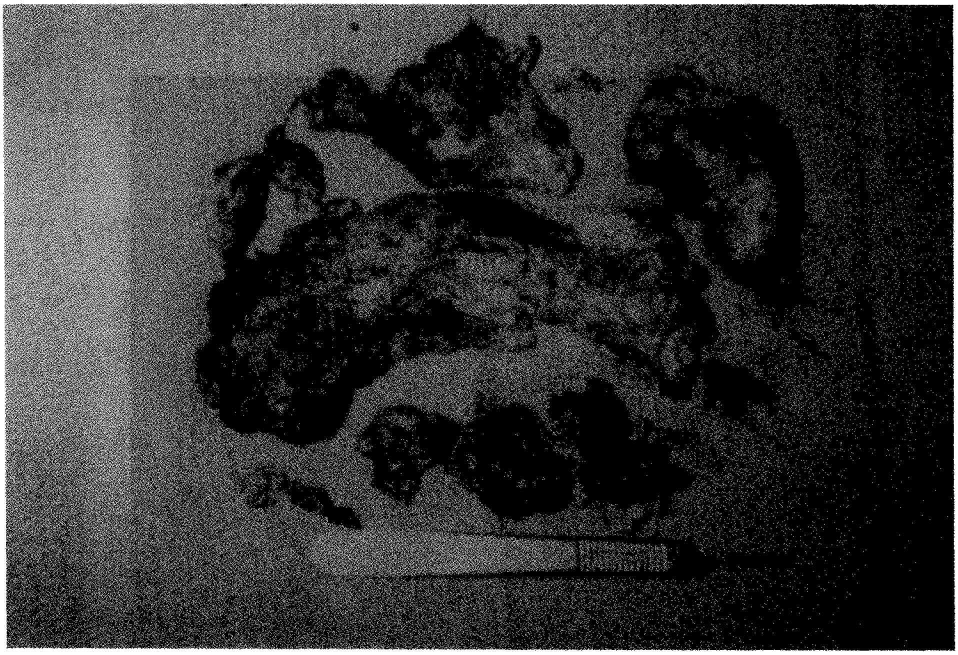

**Figure 2** Necrotic retroperitoneal, peripancreatic adipose tissue debrided on the 39th post-transplant day. This was the initial debridement.

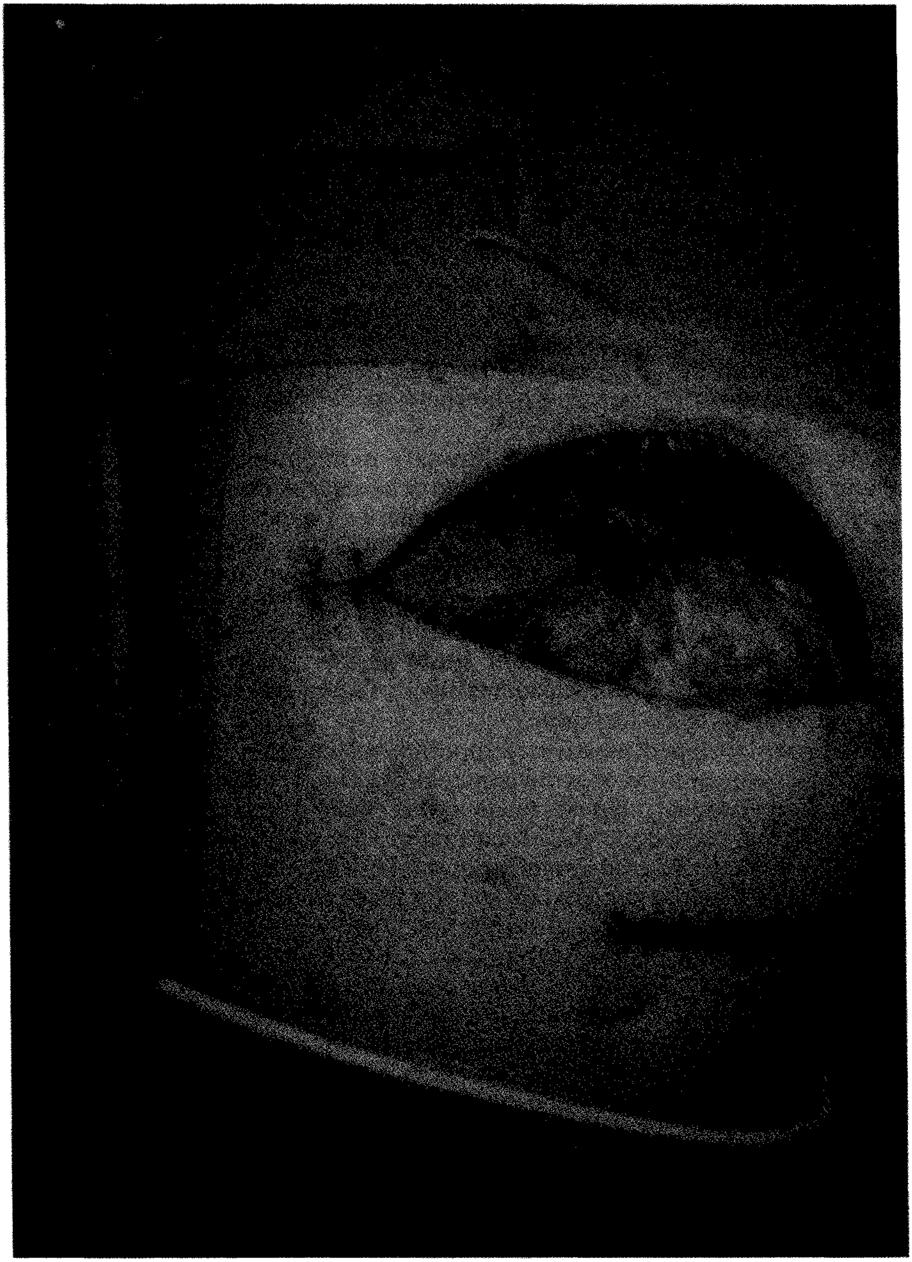

**Figure 3** The appearance of the open bilateral subcostal incision on the 23rd day after the initial debridement. Healing has been impaired. Through the sutured incision, inferior to the open wound, a jejunostomy had been performed for feeding. The small jejunostomy tube can be seen exiting the left flank.

resumed. Eighteen months after onset of the pancreatitis, ERCP revealed normal pancreatic and common bile ducts. His pancreatic endocrine and exocrine functions remain clinically stable three and a half years after the attack of pancreatitis.

### DISCUSSION

Acute pancreatitis following renal transplantation was first described by Starzl in 1964[6,7]. Since then, numerous reports have appeared. The incidence of acute pancreatitis following renal transplantation ranges from 2% to 7%[8]. A number of contributing etiological factors have been proposed in the renal transplant patient: surgical trauma, corticosteroids (especially during pulse therapy for rejection), chronic renal failure with its associated hyperparathyroidism, autoimmune disease, and viral infections[5,9,10]. Azathioprine has long been considered a major causative agent of pancreatitis in renal transplant recipients[11,12]; however, a recent study by Frick, *et al.*, disputes this assertion[13,14].

Cyclosporine is widely used as the primary immunosuppressive agent in both renal and pancreatic transplants. Controversy exists concerning the role cyclosporine plays as a causative factor in the pathogenesis of pancreatitis; Yoshimura[15] has shown that cyclosporine suppresses both the endocrine and exocrine functions in the pancreas. The same study reports that there was a significantly higher incidence of pancreatitis in those recipients treated with a cyclosporine and prednisone immunosuppressive regimen when compared to an azathioprine and prednisone regimen. In contrast, another recent study has shown no statistical difference between the incidence of acute pancreatitis in renal allograft recipients receiving either azathioprine or cyclosporine[13].

Our patient developed massive retroperitoneal necrosis around the pancreas, typical of the severest form of acute pancreatitis. The fact that most of the necrotic tissue is retroperitoneal adipose tissue rather than pancreas, *per se*, had been previously demonstrated in this department. An anatomically well preserved pancreatic duct may often be demonstrated following recovery of the patient[1].

The diagnosis of acute pancreatitis was not made initially although the hemodynamic instability and acute renal insufficiency and unusually low serum calcium levels should have provided an adequate alert. Secondary infection of the peripancreatic necrosis evolved early. The risk of pancreatic abscess formation is known to be higher in the immunocompromised renal transplant recipient. Thus, our patient combined the most life-threatening aspects of acute pancreatitis; extensive peripancreatic necrosis and early secondary infection.

Earlier reviews by Johnson and Nabseth (1970)[3], Fernandez and Rosenberg (1976)[4], and Burnstein, *et al.* (1982)[5], reported mortality rates from acute pancreatitis in renal transplant recipients of 50%, 70%, and 70%, respectively. Few authors, except Burnstein[5] and Corrodi[8] have distinguished between the more frequent, relatively benign interstitial edema of the pancreas (mortality $<10\%$) and the more malignant necrotizing pancreatitis represented by our patient. Our review of recent case reports reveals that among those deaths of renal transplant recipients that were attributed to acute pancreatitis greater than 88% (24 of 27) of the deaths were due to the necrotizing form as diagnosed by CT scan, exploratory laparotomy or postmortem. The other 12% died of the less malignant interstitial disease and/or pancreatic pseudocysts. Ten of 27 cases of necrotizing pancreatitis were undiagnosed until found at autopsy[3–5,8,10,15–21].

Important to our patient's survival was the thorough initial debridement of the retroperitoneum, the open packing of the large bilateral subcostal incision, and the primary establishment of a jejunal feeding tube via a separate abdominal incision. Although Beger[22] has reported significant progress in the treatment of acute peripancreatic necrosis by debridement and catheter irrigation, we believe that in the immunosuppressed patient, the wide open drainage may have proven lifesaving[23].

### SUMMARY

Necrotizing pancreatitis is a rare but life-threatening complication in renal transplantation. It can be detected by serial clinical examinations, serum amylase or lipase measurements, and by CT or ultrasound scans. As a complication of renal transplantation, it has led to very high mortality rates (50–100%). The initial treatment today is supportive but necrosectomy and open drainage may be needed when the patient's condition fails to improve. Sixteen necrosectomies with open drainage and irrigation were required in our patient. The combination of massive peripancreatic necrosis with immunosuppression creates a major surgical challenge. This experience indicates that survival of the patient with maintenance of function of the allografted kidney is a realistic goal.

### *References*

1. Howard, J.M. and Wagner, S.M. (1989) Pancreatography after recovery from massive pancreatic necrosis. *Ann. Surg.*, **209**, 31–35
2. Howard, J.M. (1989) Delayed debridement and external drainage of massive pancreatic or peripancreatic necrosis. *Surg., Gyn. Obstet.*, **168**, 25–29
3. Johnson, W.C. and Nabseth, D.C. (1970) Pancreatitis in renal transplantation. *Ann. Surg.*, **171**, 309–314
4. Fernandez, J.A. and Rosenberg, J.C. (1976) Post-transplantation, pancreatitis. *Surg. Gyn. Obstet.*, **143**: 795–798
5. Burnstein, M., Salter, D., Cardella, C. *et al.* (1982) Necrotizing pancreatitis in renal transplantation patients. *Can. J. of Surg.*, **25**(5), 547–549
6. Starzl, T.E., Marchioro, T.L., Dickinson, T.C. *et al.* (1964) Technique of renal transplantation. *Arch. Surg.*, **89**, 87–104
7. Starzl, T.E. (1964) *Experience in renal transplantation*. Philadelphia: W.B. Saunders
8. Corrodi, P., Dnoblauch, M., Binswanger, U. *et al.* (1975) Pancreatitis after renal transplantation. *Gut*, **16**, 285–289
9. Robinson, D.O., Alp, M.H., Grant, A.K. *et al.* (1977) Pancreatitis and renal disease. *Scand. J. Gastroent.*, **12**, 17–20
10. Fernandez-Cruz, L., Targarona, E.M., Cugat, E. *et al.* (1989) Acute pancreatitis after renal transplantation. *Br. J. Surg.*, **76**, 1132–1135
11. Mallory, A. and Kern, F. (1980) Drug-induced pancreatitis: A critical review. *Gastroenterology*, **78**, 813–820
12. Scarpelli, D.G. (1989) Toxicology of the pancreas. *Toxicol. Appl. Pharmacol* , **101**, 534–554
13. Frick, T.W., Fryd, D.S., Sutherland, D.E. *et al.* (1987) Hypercalcemia associated with pancreatitis and hyperamylasemia in renal transplant recipients. *Am. J. Surg.*, **154**, 487–489
14. Frick, T.W., Fryd, D.S., Goodale, R.L. *et al.* (1991) Lack of association between azathioprine and acute pancreatitis in renal transplantation patients (letter). *Lancet*, **337**, 251–252
15. Yoshimura, N., Nakai, I., Ohmori, Y. *et al.* (1988) Effect of cyclosporine on the endocrine and exocrine pancreas in kidney transplant recipients. *Am. J. Kidney Dis.*, **12**(L1), 11–17

16. Zisbrod, Z., Scahanzer, H., Harimov *et al.* (1981) Pancreatitis in renal transplantation recipients: A report of three cases. *Mt. Sinai J. Med.*, **48**(2), 131–132
17. Taft, P.N., Jones, A.C., Collins, G.M. *et al.* (1978) Acute pancreatitis following renal allotransplantation. A lethal complication. *Am. J. Dig. Dis.*, **23**, 541–544
18. Tilney, N.L., Collins, J.J., Jr. and Wilson, R.E. (1966) Hemorrhagic pancreatitis. A fatal complication of renal transplantation. *NEJM*, **274**, 1051–1057
19. Renning, J.A., Warden, G.D., Steven, L.E. *et al.* (1982) Pancreatitis after renal transplantation. *Int. Surg.*,, **67**, 279–280
21. Woods, J.E., Anderson, C.F., Frohnert, P.P. *et al.* (1972) Pancreatitis in renal allografted patients. *Mayo Clin. Pro.*, **47**, (Mar) 193–195
22. Beger, H.G. (1991) Operative management of necrotizing pancreatitis — necrosectomy and continuous closed postoperative lavage of the lesser sac. *Hepatogastroenterology*, **38**, 129–133
23. Bradley, E.L. 3rd (1991) Operative management of acute pancreatitis: Ventral open packing. *Hepatogastroenterology*, **38**, 134–138

*(Accepted by S. Bengmark 10 September 1992)*

